# Life in a dark biosphere: a review of circadian physiology in “arrhythmic” environments

**DOI:** 10.1007/s00360-016-1000-6

**Published:** 2016-06-04

**Authors:** Andrew David Beale, David Whitmore, Damian Moran

**Affiliations:** 1 Department of Cell and Developmental Biology, Centre for Cell and Molecular Dynamics, University College London, 21 University Street, London, WC1E 6BT UK; 2 Plant and Food Research, Seafood Technologies Group, Nelson, New Zealand

**Keywords:** Circadian clock, Physiology, Arrhythmic, Cave, Subterranean, Evolution

## Abstract

Most of the life with which humans interact is exposed to highly rhythmic and extremely predictable changes in illumination that occur with the daily events of sunrise and sunset. However, while the influence of the sun feels omnipotent to surface dwellers such as ourselves, life on earth is dominated, in terms of biomass, by organisms isolated from the direct effects of the sun. A limited understanding of what life is like away from the sun can be inferred from our knowledge of physiology and ecology in the light biosphere, but a full understanding can only be gained by studying animals from the dark biosphere, both in the laboratory and in their natural habitats. One of the least understood aspects of life in the dark biosphere is the rhythmicity of physiology and what it means to live in an environment of low or no rhythmicity. Here we describe methods that may be used to understand rhythmic physiology in the dark and summarise some of the studies of rhythmic physiology in “arrhythmic” environments, such as the poles, deep sea and caves. We review what can be understood about the adaptive value of rhythmic physiology on the Earth’s surface from studies of animals from arrhythmic environments and what role a circadian clock may play in the dark.

## Introduction

Most of the life with which humans interact is exposed to highly rhythmic and extremely predictable changes in illumination that occur as our rotating planet orbits a star in an elliptical manner. This daily light–dark cycle has a profound impact on the physiology and behaviour of the animals and plants that live and have evolved in this environment, with most, if not all, plants, animals and microbes at the Earth’s surface possessing a complex time-resolved ecology and physiology that directly or indirectly is synchronised with the light and heat given off by the sun. Yet these animals and plants reside within only a thin layer of the biosphere, and outside this thin layer, the effect of the sun is much weaker. The attenuation of sunlight through the ground is extremely rapid, with an unbroken strata of rock, soil or sand effectively absorbing all light within 10 cm of the surface (Tester and Morris 1987). The daily warming effect of the sun below ground is evident down to approximately 1 m depth, and annual variation in temperature can be experienced up to 20 m deep (Geiger et al. 2003). Below this depth, the soil and rocks are under the influence of geological processes and are relatively stable in temperature. In clear oceanic waters sunlight is reduced to starlight radiance intensities by 600–700 m depth, and no daylight can penetrate beyond approximately 1000 m (Warrant and Locket 2004). Beyond a few metres depth the sea does not experience daily variation in temperature (Kawai and Wada 2007), and annual variation in temperature is absent beyond 1000 m (Talley et al. 2011). The rhythmicity created by the daily changes in sunlight is, therefore, only experienced by organisms living in a band between the troposphere and 1000 m below the sea surface or 20 m deep into the land. However, the biosphere extends beyond this rhythmic band. Intraterrestrial life extends down at least 5 km (Pedersen 2000), and animals are found even in the deepest oceans, including Challenger Deep, 11 km below the sea surface (Gallo et al. 2015). The biosphere is, therefore, dominated by dark, largely “arrhythmic” habitats, and in terms of biomass, most of life on earth resides in places isolated from the direct effects of the sun (Whitman et al. 1998).

What is life like for organisms that reside away from the direct influence of the sun? This is a difficult question to answer given our limited ability to access the dark biosphere, but a reasonable starting point is to consider what we know about patterns of physiology and ecology in the light biosphere, and then imagine what would happen if there was no light or warmth from the sun. One immediate consequence of a shift into perpetual darkness is an inability to photosynthesize, which restricts the distribution of a substantial fraction of life to the photic zone. Primary production and food webs below the Earth’s surface are strikingly different to that which we are familiar with as a consequence of this dramatic change in energy flow through the environment. In addition, without solar light, high resolution vision is no longer possible, and unless there is a biological source of light, other senses must be relied upon for interpreting the environment. Sensory systems are consequently another major area of change for adaptation to life below the Earth’s surface.

Probably one of the least understood consequences of life in the dark biosphere is what it means to live in an environment of low or no rhythmicity and how this affects rhythmic physiology. This review sets out to discuss rhythmic physiology in animals from arrhythmic environments and why this is valuable in our understanding of the importance of rhythmic physiology on the surface. The review will start by summarising the current state of understanding about rhythmic physiology in animals to give an interested reader not familiar with biological rhythm research a primer (“An introduction to rhythmic physiology in animals”). As plants and other photosynthetic organisms are not a part of the dark biosphere we have not included them in this discussion, and while microbes are certainly the largest biomass contributor to the deep sea and underground environments, we have focussed on animals to fit within the scope of the journal. The following section describes methods used to study rhythmic physiology in animals, highlighting which are appropriate for studying animals from arrhythmic environments, especially for use in field studies. The next section reviews the research to date on rhythmic physiology in arrhythmic environments and the last section discusses what can be gained in our understanding of the evolution of physiological rhythmicity by comparing animals from rhythmic and arrhythmic habitats.

## An introduction to rhythmic physiology in animals

**Fig. 1:**
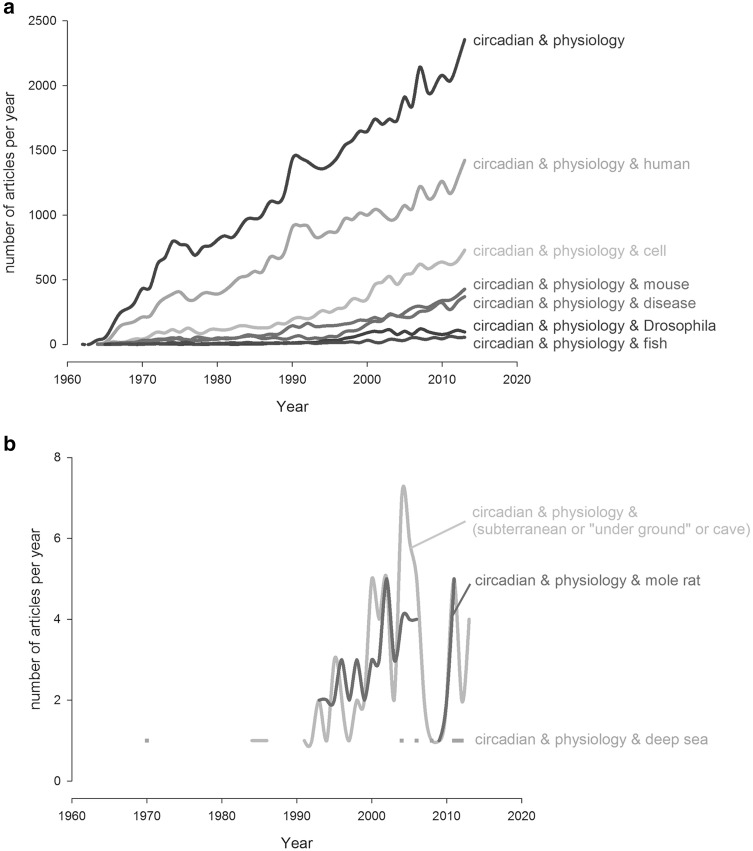
Article publication rate on topics associated with circadian physiology (source: PubMed, http://www.ncbi.nlm.nih.gov/pubmed). **a** and **b** present publication rates on different scales

Though our understanding of rhythmic physiology is expanding rapidly, it is based on studies of relatively few organisms, most of which live within the direct influence of the sun. The most studied form of rhythmic physiology is that of circadian (*circa*: approximately; *diēs*: day) periodicity, probably because this period is associated with the most marked variation in physiology in humans and many of the animals we interact with. The publication rate of studies investigating ‘circadian’ and ‘physiology’ have increased steadily since the early 1960s with more than half of these studies focusing on human biology (Fig. 1a) and an increasing awareness of the role of circadian physiology in health and disease (Fig. 1a). Other clock studies have looked at the well-studied biological models, *Mus musculus* and *Drosophila melanogaster* (Fig. 1a). *Drosophila* mutants with odd behavioural patterns were particularly important in helping to unravel the molecular clock in the early 1970’s (Konopka and Benzer 1971). These models offer the same benefits to rhythmic physiology research as they do in other fields of biology: the ease of manipulating experimental conditions in large replicated groups; the generation of “knock-out” models to study gene-level effects; and the availability of automated equipment and tools to record physiology and behaviour. Fish are an increasingly studied group of animals in clock biology (Fig. 1a), and allow the investigation of a more decentralised clock arrangement compared to other vertebrates (Tamai et al. 2005).

Like many maturing fields of biology, the research is diversifying away from laboratory-based studies on a few species to an ever expanding range of organisms across different habitats. Yet studies of species that live away from the sun are a very small fraction of rhythmic physiology research. There are only a few publications every year (0–7) that investigate the circadian physiology of organisms in caves or underground (Fig. 1b), and many of those reports have focussed on mole rats, a group of subterranean rodents with diminished visual capacity. Mexican cavefish (*Astyanax mexicanus*) are an increasingly studied subterranean species well suited to investigating adaptation of rhythmic physiology to cave life but while many studies have focused on the proximate and ultimate causes of eye loss, comparatively fewer have been on the rhythmic biology of this species. There have been very few studies of rhythmic physiology in the deep sea (Fig. 1b), probably due to the challenging nature of obtaining live samples from depths beyond the photic zone.

### The molecular circadian clock

**Fig. 2:**
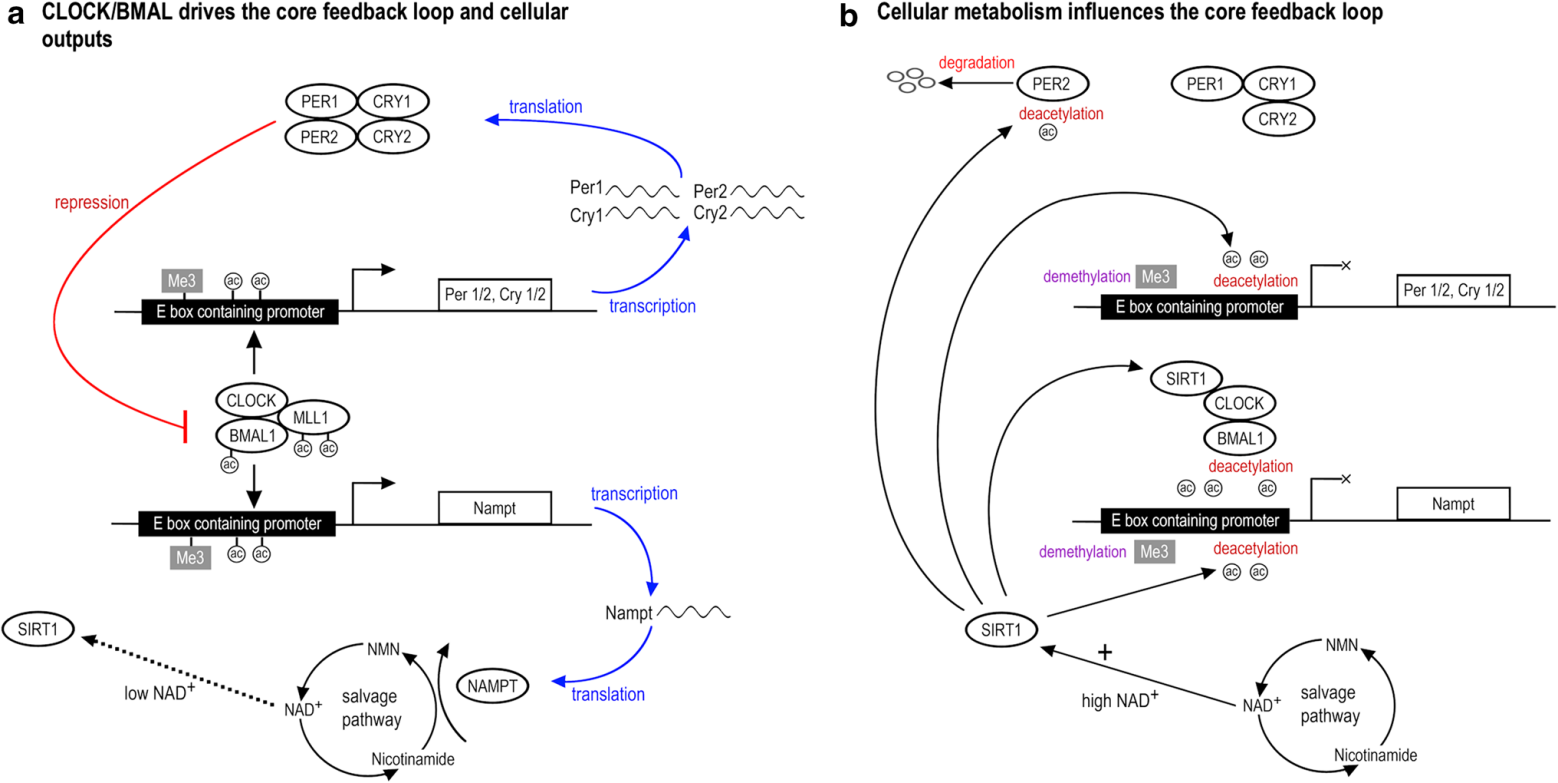
The circadian clock core feedback loops interact with cellular metabolism. **a** In the core feedback loops, rhythmic expression of PERIOD (PER1, PER2 and PER3) and CRYPTOCHROME (CRY1 and CRY2) proteins are produced from the rhythmic activation of *Period* (*Per1*, *Per2* and *Per3*) and *Cryptochome* (*Cry1* and *Cry2*) by CLOCK and BMAL1. PER and CRY proteins form complexes that subsequently translocate back to the nucleus to inhibit the CLOCK:BMAL1 activity, thus closing the loop. CLOCK and BMAL1 also activate the rhythmic expression of many other genes within the cell imparting rhythmicity on many aspects of cellular physiology, such as *Nampt*, the rate limiting enzyme in the NAD salvage pathway. **b** The NAD salvage pathway has a direct influence on the NAD+-dependent deacetylase, SIRT1. SIRT1 directly interacts with the core circadian clock elements in three major ways: (1) through deacetylating PER2, promoting its degredation (Asher et al. 2008); (2) through its effect on circadian chromatin remodelling through its deactylase action on H3K9 and H3K14 in preferential sites of CLOCK’s own acetylase activity (Doi et al. 2006; Nakahata et al. 2008). (3) Through deacetylating, modifying CRY1-mediated repression of the CLOCK/BMAL1 complex (Hirayama et al. 2007; Nakahata et al. 2008). SIRT1 also influences H3K4 trimethylation through circadian deacetylation of MLL1, causing a reduction in transcriptionally active chromatin (Aguilar-Arnal et al. 2015). Since one CLOCK target is *Nampt*, in this way, the NAD-CLOCK accessory loop via SIRT1 is closed (Nakahata et al. 2009; Ramsey et al. 2009)

Biological rhythmicity studies have shown that at the heart of rhythmic physiology is a genetic mechanism known as the circadian clock that is broadly distributed throughout the biological realm (Young and Kay 2001). This clock has been found to profoundly influence many aspects of microbial, plant and animal physiology, and helped surface-dwelling organisms evolve from a purely demand driven or reactive physiology to one which is modulated in anticipation of predictable events. The circadian clock is principally based on transcription–translation feedback loops (TTFL), where proteins from clock genes directly or indirectly affect the expression rate of the genes from which they were transcribed (Fig. 2). This arrangement results in oscillations of gene expression of roughly 24 h. In addition to being robust to perturbations in the performance of individual components (e.g., Debruyne et al. 2007a; Fan et al. 2007; Liu et al. 2008), the molecular clock is also temperature compensated, crucial for the maintenance of accurate periods in the varying temperature environments (Huang et al. 1995).

The relative importance of the different components of the clock has gradually been uncovered using gene “knock-out” models and studying animals with naturally occurring mutations. Though the component proteins are not homologous between plants, yeast and animals, the roles and their organisation into TTFLs are conserved. In mammals, heterodimerisation of two basic helix-loop-helix proteins, CLOCK and BMAL1, enables the binding of the complex to E-box elements in promoters. Binding activates expression and, PERIOD (PER1, PER2) and CRYPTOCHROME (CRY1, CRY2) proteins are produced from the rhythmic activation of *Period* (*Per1*, *Per2*) and *Cryptochome* (*Cry1*, *Cry2*) genes. Over time, PER and CRY proteins accumulate in the cytoplasm, forming complexes that subsequently translocate back to the nucleus to inhibit the CLOCK:BMAL1 transactivation and thus closing the loop (Fig. 2a). This comprises the core TTFL loop.

However, the overall mechanism is far more complex: there is a high level of redundancy and numerous peripheral modifiers. Redundancy in the mechanism can be found in the paralogous genes, where the loss of one paralogue can compensated by another. This was clearly demonstrated by a knockout of *Clock* in mice, which had no significant consequence on the overall pattern of clock gene expression or rhythmic behaviour (Debruyne et al. 2006). It was later shown that in mammals, CLOCK and NPAS2 have overlapping roles in the master clock (Debruyne et al. 2007a), meaning a single knockout of a core clock gene has little effect, though this compensation is tissue specific and NPAS2 is not able to restore function in the periphery (Debruyne et al. 2007b). Genetic redundancy is present in all animal clock mechanisms, and is particularly extreme in fish, with their numerous whole genome duplications (Postlethwait et al. 2004; Wang 2008a, b, 2009).

Whilst the TTFL forms the canonical core of the circadian clock, post-translational regulation is highly important. In fact, one of the most famous mammalian circadian mutants, the *tau* hamster, is a mutant of a posttranslational modifier, the casein kinase CK1ε, which phosphorylates PER (Ralph and Menaker 1988; Lowrey et al. 2000; Meng et al. 2008). Casein kinase mutations also lead to *Drosophila* *doubletime* (Kloss et al. 1998; Price et al. 1998) and human familial advanced sleep phase syndrome (FASPS) (Xu et al. 2005), and are a key element in the circadian system of zebrafish (Smadja Storz et al. 2013). Components in the circadian mechanism can also undergo acetylation, methylation, SUMOylation and glycosylation, providing layer upon layer of regulatory fine-tuning. Post-translational mechanisms modulate clock protein turnover (e.g. CK1—Meng et al. 2008), intracellular localization (e.g., SUMOylation of BMAL1—Lee et al. 2008), and transcriptional activity (e.g., glycosylation of CLOCK and PERIOD—Kaasik et al. 2013) and thus play key roles in the overall running of the oscillator (for an excellent review on post-translational modifications of clock proteins see Mehra et al. 2009).

Interaction with post-translational modification systems shows that the clock does not operate in isolation from cellular physiology, and likewise cellular physiology is not in isolation from the circadian clock. A particularly intertwined example of this crosstalk is related to NAD biosynthesis, where metabolic pathways influence the circadian clock and the clock influences metabolism (Fig. 2b; Nakahata et al. 2009; Ramsey et al. 2009). The importance of metabolic and cytosolic cycles in circadian timing is also seen in the non-transcriptional clock mechanisms, such as the Kai clock of cyanobacteria and the peroxiredoxin clock of human blood cells, unicellular algae and other organisms (Tomita et al. 2005; O’Neill and Reddy 2011; O’Neill et al. 2011; Edgar et al. 2012). Though these mechanisms are able to generate circadian oscillations in the absence of the TTFL, it is likely that both the transcriptional and non-transcriptional mechanisms are important in fulfilling the requirements of coordinating timing of cellular physiology (Edgar et al. 2012; Reddy and Rey 2014). This considerable mechanistic rhythmicity is realised downstream in the fact that at least 10 % of the transcriptome oscillates (Panda et al. 2002; Miller et al. 2007), with CLOCK-dependent oscillatory expression evident in a diverse array of metabolic pathways including the amino acid, carbohydrate, lipid, nucleotide, and xenobiotic pathways (Eckel-Mahan et al. 2012). The circadian clock and cellular physiology are clearly tightly linked (for review see Reddy and Rey 2014).

### Entraining to external cues

This molecular mechanism is not a stand-alone time keeper, rather it is modified or reset according to external cues such as light, temperature and food availability. This ability to tune or entrain to external cues is the defining feature of a biological clock. When the rhythmicity of these cues or zeitgebers (from the German, “time-giver”) is removed and the organism is placed under constant conditions, internal biological time becomes evident: the clock and physiology associated with it ‘free runs’, with a ‘free-running period’ of minutes to hours longer or shorter than the period the organism was previously entrained to (hence the term *circa* in circadian).

Many types of zeitgebers have been found to synchronise circadian clocks, from abiotic factors such as light, temperature, sound and mechanical disturbance to biotic factors such as food availability and feeding, social contact and the activity of other organisms (Refinetti 2005). Light intensity is the most easily detectable and reliable source of information that animals can use to entrain their circadian rhythms, and is the most obvious difference between rhythmic and arrhythmic habitats. Photoreception is widespread throughout the biological realm, and non-directional irradiance photoreception for day/night detection likely drove the early evolution of photoreceptors (Nilsson 2013; Lamb 2013). Long-integrating photoreceptive cells that entrain the clock persist in most animals, and can be found as distinct cells in the retina, brains and parietal eyes (‘third’ or ‘pineal’) of many vertebrates (Berson et al. 2002; Vigh et al. 2002; Berson 2003; Bertolucci and Foà 2004) and as optic or non-optic components of the clock network in invertebrates (Saunders 2002; Land and Nilsson 2012). Within these cells are expressed non-visual photopigments, with *Melanopsin* and *cryptochrome* the major circadian photopigments in mammals and *Drosophila,* respectively, (Stanewsky et al. 1998; Lucas et al. 2001; Hattar et al. 2003). Melanopsin shares greater homology with invertebrate opsins than visual opsins, resembling invertebrate opsins in its tyrosine counterion, third cytoplasmic loop, and extended C terminus (Provencio et al. 1998; Bellingham and Foster 2002; Hankins et al. 2008). Multiple non-visual opsin genes exist in non-mammalian vertebrates, with 32 genes including 5 *melanopsin* genes identified in zebrafish, though none have yet been linked mechanistically to circadian entrainment (Davies et al. 2011, 2015).

In mammals, the master circadian clock is necessary and sufficient to orchestrate the timing of circadian rhythms in the rest of the body: transplantation of the SCN between mutant hamsters with different periods transfers the donor period to the recipient animal (Ralph et al. 1990). The SCN receives light input from specialised photosensitive retinal ganglion cells in the eyes via the retinohypothalamic tract (Moore and Eichler 1972; Berson et al. 2002). The master clock–peripheral clock arrangement is not as clear cut outside of the mammals. A subset of brain neurons act like a central pacemaker in *Drosophila* and generate circadian rhythms of behaviour (Renn et al. 1999; Hardin 2005), however, unlike mammals, *Drosophila* peripheral cellular circadian clocks can be directly entrained by light, meaning that environmental cycles can be directly coordinated with cellular physiology in the absence of specialised clock centres (Plautz et al. 1997). This decentralization of the clock organisation is also observed in other clock models such as zebrafish (Whitmore et al. 1998, 2000). In fact, a central circadian pacemaker is yet to be localised in zebrafish and the brain is globally responsive to light, reflecting a high degree of direct light sensitivity in this animal as a whole (Whitmore et al. 1998; Moore and Whitmore 2014; Davies et al. 2015).

While light is probably the dominant zeitgeber for most animals, temperature can also entrain rhythms, though its importance as a zeitgeber depends on clock hierarchy and whether the animal is poikilothermic or homoeothermic (for review see Rensing and Ruoff 2002). Temperature cycles strongly entrain rhythms of eclosion in flies (Zimmerman et al. 1968), and behavioural rhythms in poikilotherms such as lizards, flies and fish (Hoffmann 1969; Wheeler et al. 1993; Glaser and Stanewsky 2005; Lahiri et al. 2005; Sehadova et al. 2009). While temperature can entrain behavioural rhythms in homeotherms such as mice and squirrel monkeys (Aschoff and Tokura 1986; Refinetti 2010), it is only a weak zeitgeber, and the master clock in the suprachiasmatic nucleus (SCN) is resistant to temperature entrainment (Buhr et al. 2010; Refinetti 2015). It is likely that in homoeotherms temperature cycles serve as internal zeitgeber signals for downstream peripheral oscillators (Brown et al. 2002; Buhr et al. 2010).

Thus, the circadian clock is a highly complex mechanism, not just consisting of a stand-alone transcription–feedback loop but also of numerous accessory loops (which add robustness and interconnectivity within the cell) and an ability to entrain to relevant environmental cues at different time scales. The clock mechanism is vital not just for the rhythmic coordination with the external world, but also for the rhythmicity of the cellular environment in all its guises. It is clear that circadian clock and physiology are tightly intertwined and any disruption to the system is likely to have a large impact on the organism.

## Methods to study circadian physiology

Circadian physiology is difficult to study for two reasons. First, it is generally difficult studying physiology as the processes of interest tend to occur within a whole organism. Outward manifestations of physiology such as behaviour or morphology can be used to infer what is happening inside the organism, but much of the research on circadian physiology aims to trace the action of metabolic processes to the internal clocks, which tends to require a reductionist approach towards measurement of metabolite flux, gene expression, post-translational modifications and so on. Tools continue to be developed to study a broad array of variables simultaneously (e.g., metabolomics and gene microarrays), or to report the flux of physiological processes in living organisms (e.g., fluorescent reporter genes, implantable biometric devices and microcannulation) in an effort to make sense of the multitude of interactions between genes, gene products, metabolites and hormones.

The second challenge of circadian physiology research is developing methods to study a process of interest repeatedly over time. Single point sampling has limited value, as circadian physiologists are interested in repeated measures over hours, days, months and years. There are two approaches that can be used to develop a physiological profile over time: the first involves taking samples from multiple individuals (or tissues or cells) at different time points and plotting an average value against time, and the second involves resampling the same individuals over time. The latter method is probably optimal as it reduces the overall error in the study (Walter et al. 2014); however, obtaining serial samples from the same individuals can be difficult. The degree of intra-individual variation in rhythmic data can be considerable, especially at higher levels of biological organisation such as circadian behaviour or whole body energy metabolism, probably because there are so many components that interact to generate higher order physiological rhythms. At reduced levels of biological complexity, such as at the single cell level, circadian clock oscillations and physiological cycling can be remarkably uniform and robust. In mammals the SCN has very robust rhythmicity as a whole due to extensive synaptic coupling and numerous gap junctions (Yamaguchi et al. 2003; Liu et al. 2007), while cells in other tissues tend to have a lower degree of cellular connectivity and as a result a less robust physiological rhythmicity (Liu et al. 2007; Abraham et al. 2010). Thus, researchers can face significant challenges in detecting free-running physiological rhythms in whole animals due to the varying biological rhythmicity of different tissue types. In addition, a study must be constructed that controls for the many zeitgebers that clocks within these tissues may differentially entrain to (e.g., food availability, daily and seasonal light cycles, and auditory, tidal and temperature cycles). The following text discusses methods and tools that can be used to collect rhythmic physiology data, and which of those are appropriate for studying animals from arrhythmic environments.

The first group of research tools is behavioural tracking methods that allow researchers to quantify time-resolved activity intensity. These tools may be deployed in the wild or in the laboratory and, since they are non-invasive, are useful for studying species with limited population numbers and ranges, such as those found in extreme environments. These tools can take the form of motion recording using video cameras (Bailoo et al. 2010), monitored exercise equipment (e.g. running wheels for mice—Thomas et al. 2011), accelerometers mounted on the body (Ropert-Coudert and Wilson 2005), photo-cell beam interference (Barrett et al. 2001; Thomas et al. 2011), and acoustic monitoring (Bovet and Oertli 1974). Automated computer analysis software is commonly used to track and quantify activity, which, in addition to being more efficient, can also be more sensitive and objective than human analysis of behavioural patterns (Desland et al. 2014). An important consideration in video analysis of behaviour is the ability to monitor activity at low light or dark periods, which is especially important in the study of animals that normally exist in the absence of light. Infrared lighting can be used to illuminate arenas under conditions that would be perceived as dark for most animals (with the exception of some snakes and insects), and infrared backlighting often helps contrast objects under all lighting conditions (Bailoo et al. 2010).

The next group of tools to study rhythmic physiology is that which facilitate the measurement of metabolite flux or physiological status in living animals. These tools include implanted devices to measure body temperature (Minors et al. 1996), nerve activity (Barrett et al. 2001) and blood pressure and flow rate (Westgate et al. 2008). Such devices may be wired (meaning the animal is tethered) or wireless, with key considerations being the ability to power the device, the size of the implant and data transmission capacity (or logger retrieval). Time-resolved measurement of hormone flux can be achieved through serial blood sampling via an implanted cannula and microdialysis (Drijfhout et al. 1996; Solberg et al. 2001), or via the sampling of bodily fluids such as saliva (Shirakawa et al. 2004) and urine (Feldmann et al. 1989). It is possible to calculate hormone flux in aquatic animals through measurement of hormones excreted into the water (Ellis et al. 2007). With the exception of excreted hormone measurement in aquatic animals, these tools are relatively invasive procedures and as such may not be very useful in lab or field studies of extremophiles.

Another category of tools to study metabolite flux is the methods to quantify metabolic rate. Direct calorimetry measures the rate of heat production, and can be used to monitor the time-resolved energy use of terrestrial animals inside a thermally insulated chamber (Berman and Meltzer 1978). The most common method to quantify aerobic energy output in aquatic animals is to monitor oxygen consumption in a chamber (Steffensen 1989). The oxygen consumption rate of aerial breathers can also be used to measure metabolic rate over time (Refinetti 2003), as can the rate of carbon dioxide excretion (Krauchi and Wirz-Justice 1994), and by comparing the two values it is possible to approximate the proportion of metabolism powered by carbohydrate versus fat catabolism (Gnaiger 1983). While it is extremely difficult to quantify the carbon dioxide excretion rate of aquatic animals accurately, instantaneous nitrogen excretion is relatively easy to measure, and when combined with oxygen consumption rates allows for the calculation of protein catabolism rates over time (Stiller et al. 2015). Quantifying the time-resolved metabolic rates of animals in the field is effectively impossible with current technology, although accelerometry is an increasingly popular proxy method for measuring energy expenditure of animals in their natural environment (Cooke et al. 2004; Payne et al. 2014).

A further set of tools and methods allows the measurement of the molecular genetic clock and time-resolved variation in the transcriptome, proteome and metabolome (Gaspar and Brown 2015). DNA microarray analysis has been important in elucidating the cyclical transcription of both clock genes and the genes downstream that are affected by the oscillator state. However, considerable effort needs to be expended to ensure that there is sufficient statistical power to confirm changes in gene expression (Straume 2004; Lee et al. 2000; Delaunay and Laudet 2002). Most studies require a serial sampling approach which can be difficult in the field, though this has not prevented them from being used in Mexican cavefish (Beale et al. 2013). The use of primary cell cultures to study rhythmic physiology can circumvent some of issues around serial sampling of multiple individuals in that subsamples of the same primary culture can be analysed over time (Earnest and Cassone 2005). However, while cell culture approaches are useful for probing the components and functioning of the molecular clock and attendant outputs (Nagoshi et al. 2005; Farnell et al. 2011), they are not able to directly inform on the instantaneous state of genes or metabolites in the tissue or organism from which the cells were harvested.

**Fig. 3:**
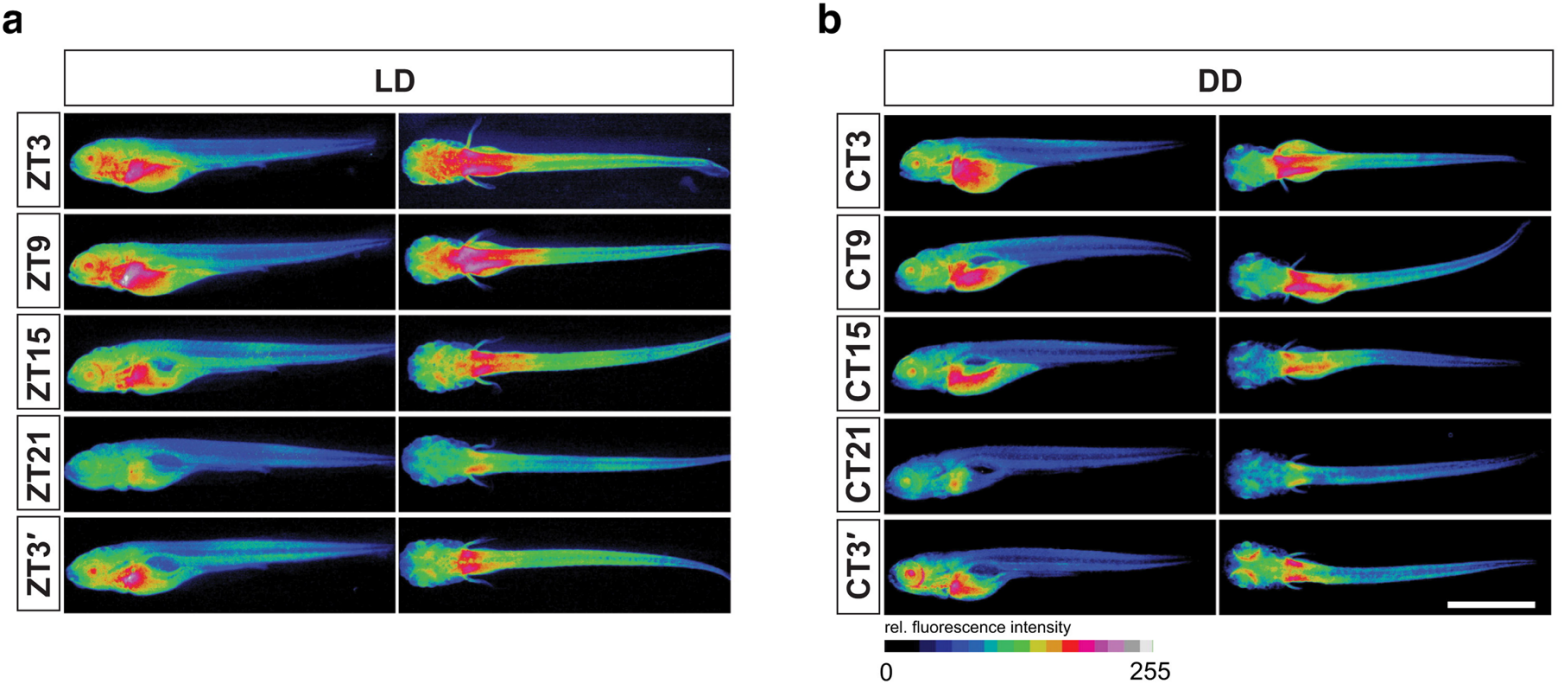
E-box reporter activity in zebrafish larvae. Luciferase expression from *tg(4xE*-*box:Luc)*, detected by immunohistochemistry with an anti-luciferase antibody and an Alexa Fluor 488-labelled secondary antibody, shows diurnal (**a**) and circadian (**b**) rhythms across all tissues of a 5 dpf larvae. Relative fluorescence intensity is shown *colour coded*. Larvae were sampled at five different time points during LD (ZT3-ZT3′; **a**) or DD (CT3-CT3′; **b**) after a 4-day entrainment period. *Scale bar* 1.0 mm. Taken from Weger et al. (2013). Reprinted from Developmental Biology, 380/2, Weger et al., Real-time in vivo monitoring of circadian E-box enhancer activity: a robust and sensitive zebrafish reporter line for developmental, chemical and neural biology of the circadian clock, 259–273, Copyright (2013), with permission from Elsevier

A final tool can be used to monitor gene expression in real time in cells, tissues and even whole animals. In the study of rhythmic physiology, this is most commonly a knock-in of a reporter construct consisting of a clock gene promoter driving a firefly luciferase gene (Fig. 3; Welsh et al. 2005). This generates dim luminescent light in synchrony with the endogenous activation of the clock gene which can be captured with a high sensitivity camera (Welsh et al. 2005; Yamazaki and Takahashi 2005; Carr and Whitmore 2005). The use of optical fibres inserted into animals carrying the luciferase reporter has allowed researchers to visualise and measure the cycling of clock genes in intact organs (Yamaguchi et al. 2001; Wilsbacher et al. 2002). The ability to measure spatiotemporal gene expression in living tissue and whole animals in this way has rapidly progressed the field of rhythmic physiology and changed our understanding about molecular clock modulation, distribution, hierarchy and links to downstream metabolic processes. While this tool is not useful for studying animals in their natural habitats, advances in DNA sequencing have improved the possibility of generating reporters for multiple clock genes in non-model organisms.

## A review of rhythmic physiology research in animals from arrhythmic habitats

The dark biosphere is the largest habitable zone on earth, with a large biomass of resident organisms. We would therefore be remiss not to study what appears to be a universal feature of life on Earth in a substantial fraction of what actually lives on the planet. What is known about rhythmic physiology in these habitats?

### Polar habitats

Polar regions, with their extreme annual variation in photoperiod, are neither constantly rhythmic nor fully arrhythmic but represent a unique challenge to the animals that live there. At latitudes above 78°N and below 78°S there are periods of the year when the sun remains below the horizon (and conversely, periods when the sun remains above the horizon) for multiple days (Berge et al. 2015). The resulting ‘polar night’ and ‘polar day’ results in organisms experiencing constant darkness and constant light for periods throughout the year. With increasing latitude, there is a corresponding decrease in amplitude of the light:dark (LD) cycle for an increasingly large part of the year and animals that live in these high latitudes may experience distinct LD cycles for less than a third of the year.

**Fig. 4:**
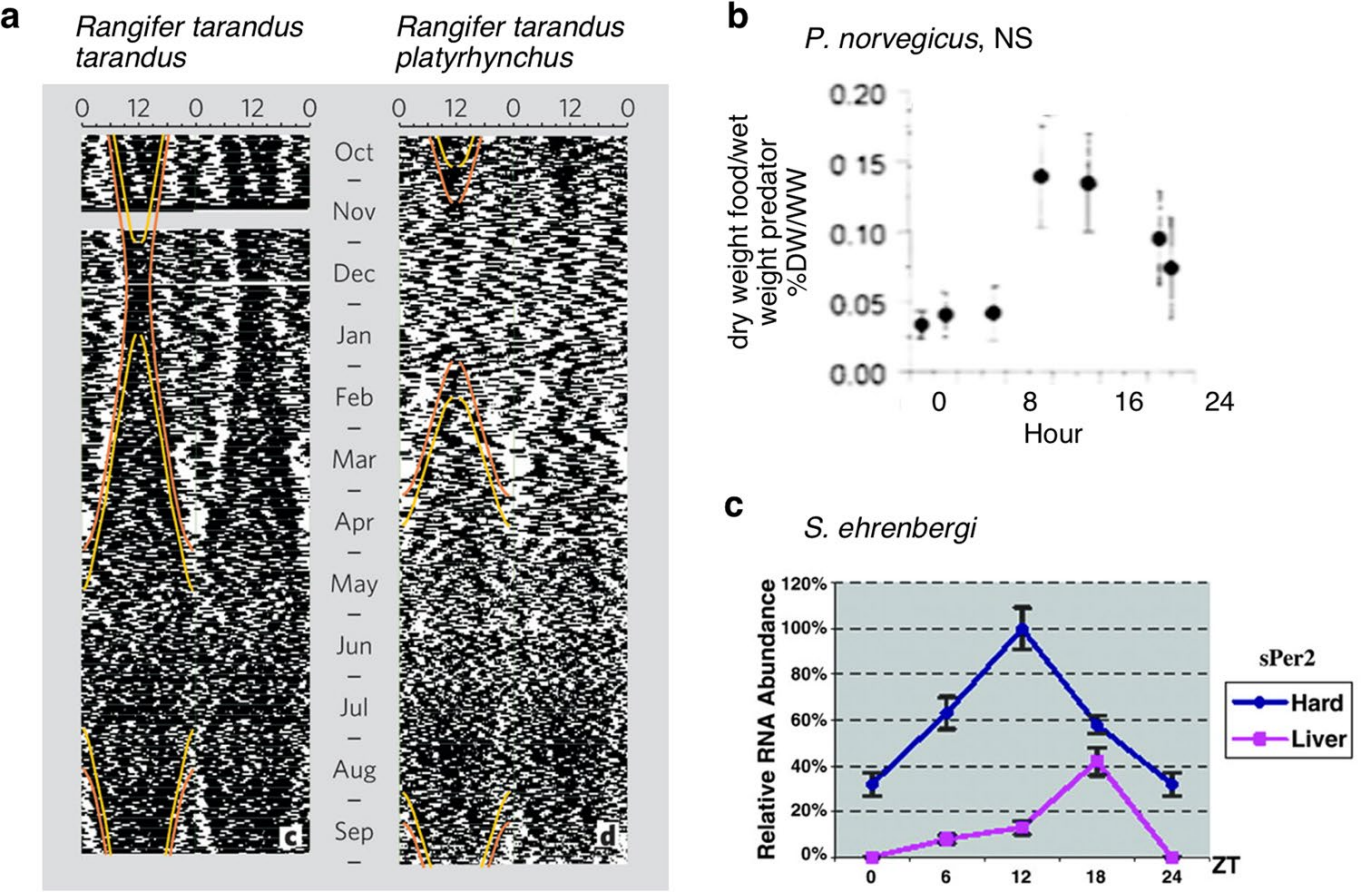
**a** Sample actograms showing patterns of activity over 1 year in sub-adult reindeer in *left*, northern Norway at 70°N (*R. t. tarandus*), and *right*, Svalbard at 78°N (*R. t. platyrhynchus*). *Lines* indicating the beginning and end of civil twilight (when light intensity is 10 lx, *orange*) and sunrise and sunset (*yellow*) are superimposed on each actogram (van Oort et al. 2005). Reprinted by permission from Macmillan Publishers Ltd: Nature, van Oort et al. 2005, copyright 2005. **b** Hints of rhythmicity in a deep-sea decapod at 1178–1240 m below sea level, *Pontophilus norvegicus*, as indicated by the variation in stomach fullness of fish caught at different times of day, however ANOVA reveals no significant difference between samples (Maynou and Cartes 1998). Copyright © 1998 Inter-Research. **c** Core clock gene rhythms in peripheral tissues of the mole rat, *Spalax ehrenbergi*, on a light–dark cycle. Here, RT-PCR reveals that *Per2* shows a high amplitude rhythm in both the harderian gland (Hard) and the liver (Avivi et al. 2002). Copyright © 2002, The National Academy of Sciences

The islands of Svalbard sit between 74° and 81°N and host two of the most well-studied resident arctic herbivore species, the Svalbard reindeer (*Rangifer tarandus platyrhynchus*) and ptarmigan (*Lagopus mutus hyperboreus*). Both animals lose daily behavioural rhythmicity during the polar day, resulting in periods of constant activity (Fig. 4a) and arrhythmic melatonin secretion, a marker of internal circadian rhythmicity (Reierth et al. 1999; van Oort et al. 2005, 2007; Stokkan et al. 2007; Lu et al. 2010). In arctic reindeer, melatonin secretion appears to be independent of circadian rhythms, as rhythms of melatonin secretion in juveniles exposed to LD cycles do not persist in constant darkness and are instead driven by acute changes in light (Stokkan et al. 2007; Lu et al. 2010). In contrast to mouse fibroblasts containing the same reporter constructs, transgenic reindeer fibroblasts carrying mouse *Bmal1* or *Per2* clock gene reporter constructs showed unstable and transient oscillations for one to two cycles with a range of periods from 19 to 31 h (Lu et al. 2010). Taken together with the absence of rhythmic melatonin secretion, these results suggest that the molecular clock that drives circadian rhythms in these polar animals may be absent, or at least very weak.

However, it is unknown whether this faithfully reflects the nature of the molecular circadian clock since these results are from cross-species reporter constructs and not compared to the clocks of temperate ungulates. While showing a similar absence of melatonin rhythms during the polar day, ptarmigans, in contrast to the reindeer, show clear daily cycles of melatonin persisting throughout the polar night, suggestive that at least some of the circadian system remains functional in these birds (Reierth et al. 1999). Persistent melatonin rhythms exist throughout the polar day conditions in willow warblers and Lapland longspurs, though absolute levels were reduced (Hau et al. 2002; Silverin et al. 2009; Ashley et al. 2014). The suppression and lack of rhythmicity in melatonin seen in the arctic summer is very likely due to the suppressive action of bright light (Wurtman et al. 1963).

One possible explanation to the diversity of behavioural and physiological rhythmicity is that circadian clock control of outputs is plastic in the unusual illumination of the polar environment. Supportive of this is the finding in two species of arctic birds of a diversity of activity patterns in constant light (entrained rhythm, ‘free-running’-like rhythm and arrhythmic) which depend on life history, sex and breeding stage (Steiger et al. 2013). This explanation requires that the clock continues to oscillate throughout the polar day, with clock control of outputs only employed when it is beneficial to do so, which depends on life history and time of year (Williams et al. 2015).

The persistence of entrainable circadian rhythms of physiology and behaviour in polar animals at different points of the year may represent some sort of halfway house between the full benefits of circadian rhythms in a rhythmic environment and the unknown role in an arrhythmic environment. In this environment, the condition of the circadian system may have more to do with the importance of circannual timers, which depend on a melatonin signal (Lincoln et al. 2006; Lu et al. 2010).

### Deep-sea habitats

The deep sea is one of the least studied habitats on earth, with just 5-7 % of the deep-sea floor having been explored (Cuvelier et al. 2014). Regions below 1000 m depth are considered to be outside the reach of sunlight (Warrant and Locket 2004) and represent an arrhythmic habitat of great interest to circadian biologists. Unfortunately, very little research on the daily rhythmic lives of deep-sea organisms has been conducted, preventing strong conclusions on the rhythmic physiology of this community. However, studies by Maynou and Cartes (1998), Wagner et al. (2007), Modica et al. (2014) and Cuvelier et al. (2014) have provided some hints on rhythmicity in the deep.

Feeding rhythms in deep-sea animals have been detected by measuring the daily variation in stomach fullness after catch (Maynou and Cartes 1998; Modica et al. 2014). Of the three deep-sea decapod species that were sampled from below 1000 m depth, one, *Pontophilus novegicus*, hints at a daily rhythm in feeding by showing a demarcation between day and night (Fig. 4b; Maynou and Cartes 1998). Similar hints are seen for three of five species of fish from the deep Balearic Basin: *Coryphaenoides mediterraneus*, *Alepocephalus rostratus*, and *Lepidion lepidion* (Modica et al. 2014). Interestingly these three fish species predominantly feed on active prey. It may be that the movements and availability of active prey at different times of day itself serves as a zeitgeber for species that live deeper than 1000 m.

Melatonin secretion has been measured in two fish, the grenadier and deep-sea eel, from the deep north-eastern Atlantic (Wagner et al. 2007). Pineal melatonin content in fish caught from trawls conducted throughout the day and night suggested rhythms of half-day periods, which, when plotted against lunar time and compared to velocity measurements in currents, was highly suggestive of lunar and tidal influence even at a depth of more than 1050 m. Wagner et al. (2007) also measured spontaneous melatonin release in pineal glands in culture, a laudable effort on board a trawler vessel. Though the data is close to the minimum requirement for autocorrelation and the peaks that emerge are not significant at *p* <0.05, the occurrence of peaks at 9–11 h in all individual cultures is weakly suggestive of an underlying biological rhythm. However, limitations in the ability of the on board culture experiments to avoid the influence of exogenous rhythms reduces the reliability of the conclusions.

Unfortunately, these studies suffer from the same experimental constraint: the difficulty in obtaining regularly spaced samples from the deep-sea trawls. A better method may be remote monitoring offered by networks of cameras fixed on the sea floor. A recent study, using video footage to analyse deep-sea activity, strengthens these tentative conclusions (Cuvelier et al. 2014). Cuvelier et al. (2014) measured the faunal density (the percentage of field of view occupied by organisms) of four invertebrate species at a hydrothermal vent over a continuous 23-day recording period. Of the four species, the siboglinid tubeworms, *Ridgeia piscesae*, showed the strongest population rhythms of activity in the circadian and half-day range. These rhythms correlated with measurements of temperature rhythms at nearby locations, again demonstrating the influence of the tide even at these depths (2200 m). It is worth noting, however, that the absence of clear rhythmicity in the other three invertebrate species at the hydrothermal vent could perhaps be due to the experimental conditions: the recording required a constant source of bright light, which inhibits rhythmic activity in other animals. The bright light could also explain the gradual decrease in fauna density during the recording due to a photophobic reaction within the camera’s field of view, such as that noted by Beale et al. (2013).

Another piece of evidence comes from the non-visual opsins, candidates for being the photoreceptors in the circadian system. While non-visual opsins remain undiscovered in the aforementioned species living in the aphotic zone, functional *melanopsin* photopigments have been discovered in a deep-sea fish, the elephant shark, *Callorhinchus milii*, which could be used for the entrainment of a circadian clock (Davies et al. 2012). Therefore, although it is difficult to assign the presence of rhythms to an internal biological clock or an acute response to the environment in many of these deep-sea studies, taken together the evidence clearly demonstrates that the deep sea is not as arrhythmic as previously thought.

### Subterranean habitats

Though light is strongly attenuated within 10 cm of surface (Tester and Morris 1987) and the daily warming effect of sun only reaches 1 m (Geiger et al. 2003), a surprising number of subterranean animals possess rhythmic physiology. The most well-studied subterranean vertebrates are the mole rats of southern Africa. The mole rats rarely, if ever, are exposed to light because they live in sealed burrow systems. These include the blind mole rat, *Spalax ehrenbergi* (Tobler et al. 1998), the naked mole rat, *Heterocephalus glaber* (Riccio and Goldman 2000), the solitary Cape mole rat, *Georhychus capensis,* and four mole rat species of the *Cryptomys* genus of South Africa (Oosthuizen et al. 2003; Hart et al. 2004; Schöttner et al. 2006). Despite the subterranean habitats, strong rhythms of activity are exhibited in all studied species (Tobler et al. 1998; Riccio and Goldman 2000; Oosthuizen et al. 2003; Hart et al. 2004; Schöttner et al. 2006) and clear rhythms in clock gene expression are seen in *Spalax ehrenbergi* which also possess the circadian photoreceptor *melanopsin* and a functional, spectrally tuned photopigment (Fig. 4c; David-Gray et al. 1998, 1999; Hannibal et al. 2002; Avivi et al. 2002, 2004). There is some suggestion that social species display weaker activity rhythms than solitary species, as a greater percentage of individuals of a solitary species are rhythmic (Hart et al. 2004). However, when the molecular clock is examined, this difference disappears. Though only half the individuals studied by Tobler et al. (1998) exhibited free-running activity rhythms in constant darkness (DD), clear rhythms of clock gene expression are observed in this species (Avivi et al. 2002, 2004). It is possible that the variation in individual activity entrainment reflects a weak coupling between the endogenous clock and locomotor output, rather than a weak circadian clock, as is possible for the arctic pectoral sandpipers and male red phalaropes (Steiger et al. 2013).

Subterranean rodents from other parts of the world have been studied but, unlike the mole rats, they spend varying amounts of time on the surface, exposing themselves to light to forage and clean (Tomotani et al. 2012). One such animal is the South American solitary burrowing rodent, *Ctenomys knightii*, commonly known as the Tuco-tuco. These rodents show circadian rhythms of activity both in laboratory conditions (Valentinuzzi et al. 2009) and when brought into the lab directly from the field (Tomotani et al. 2012), again demonstrating the presence of a circadian system in a subterranean animal. However, even pulses of light can entrain circadian clocks (Carr and Whitmore 2005), and light-sampling behaviour, even to less than 3.5 % of the total light period, is a sufficient entraining signal for nocturnal rodents (Decoursey 1986; Decoursey and Menon 1991). As such, despite their apparent subterranean existence, this species does not conform to the ‘dark’ or arrhythmic definition of subterranean, as discussed here.

Subterranean life is dominated by invertebrate and microbial life, much of which is undiscovered and unresearched (Whitman et al. 1998). Whilst invertebrates are not discussed in detail in this review, it is worth considering the subterranean origins of one of the major modal organisms in biological research, the nematode worm *Caenorhabditis elegans*. These animals were isolated from subsurface habitats, including compost, mushroom beds and garden soil (Kiontke and Sudhaus 2006). Though these animals have a very short generation time, potentially masking the detection of rhythmic physiology, an entrainable circadian clock has been detected, synchronisable by both photic and temperature signals (Kippert et al. 2002; Simonetta et al. 2009). Furthermore, several physiological variables such as food consumption, pharyngeal contractions, defecation and oxygen consumption are clock-controlled in *C. elegans*, showing daily rhythms that are sustained under constant conditions and under different entrainment conditions (Migliori et al. 2011; Eelderink-Chen et al. 2015). A number of elements of the circadian system of *C. elegans* are unknown, such as the environmental time cue responsible for entrainment in the wild and the molecular components of the clock. However, it is clear for this subterranean invertebrate at least, circadian physiology is “present and correct”.

### Cave habitats

Of all the arrhythmic habitats, caves are potentially the most arrhythmic. In addition to the absence of light and dark cycles, temperature is extremely stable, approximately equal to the mean annual temperature at surface level. Water quality in underground pools is steady with a stable concentration of dissolved inorganic compounds and a low level of organic matter and evaporation rates are low, resulting in high humidity levels (Poulson and White 1969). Though there are clear examples of strongly rhythmic animals that use caves, such as bats, constitutively cave-dwelling animals, known as troglobites, live in the depths of caves and are unable to survive outside of caves. Troglobites are the most strongly specialised cave-dwelling animals, often showing complete absence of vision and pigmentation.

Caves can be “considered natural laboratories for studying the adaptive significance of the biological clock” (Poulson and White 1969). Unfortunately, few circadian studies have been performed on cave animals and detailed comparisons of the published work are difficult due to differences in experimental conditions. However, trends can be drawn out from a selection of the strongest work, most of which examines locomotor activity rhythms. Readers are also directed to an excellent summary of the cave clock literature in relation to visual systems in Friedrich (2013).

**Fig. 5:**
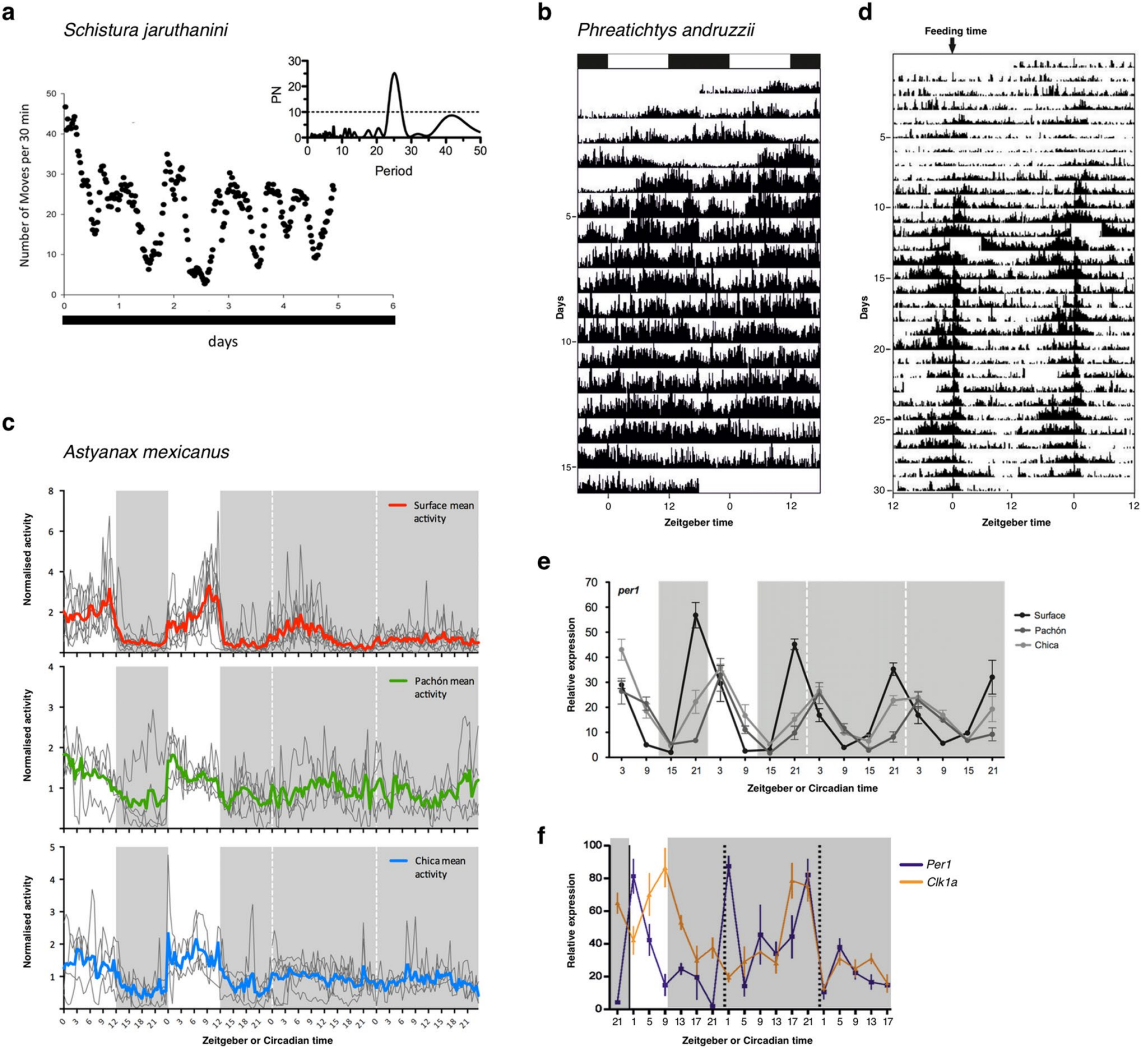
**a** The cave fish, *Schistura jaruthanini*, shows pronounced free-running locomotor rhythms after 6 months in 12:12 LD cycle. Activity was measured as the number of movements per 30 min period (*y-axis*) and was plotted over the full period of observation. Lomb-Scargle periodogram (*right*) shows pronounced rhythmicity in circadian range, typical of all but one species in Duboué et al.’s study (Duboué and Borowsky 2012). **b** A second cavefish, *Phreatichys andruzzii*, shows an absence of circadian rhythms during 12:12 LD cycles. Fish were maintained at 27 °C during recording and locomotor activity, measured by infra-red beam crossing, is double plotted on the *x-axis* (Cavallari et al. 2011). **c** Two independent populations of cavefish demonstrate an absence of circadian rhythms of activity in constant darkness after LD entrainment in *Astyanax mexicanus*, though the sister surface population is rhythmic under the same protocol. The cavefish of this species show rhythmic activity under 12:12 LD cycles (Beale et al. 2013). **d** *Phreatichthys andruzzii* cavefish, arrhythmic after LD entrainment, show circadian rhythms of locomotor activity under entrainment by an alternative zeitgeber, feeding. Fish were maintained under constant darkness and fed once a day at ZT0 and show a clear response to this daily zeitgeber. However, entrainment was not tested under constant conditions, i.e., the absence of feeding (Cavallari et al. 2011). **e** Though Pachón and Chica populations of *A. mexicanus* do not show rhythms of locomotor activity in DD after LD entrainment, an underlying *Per1* gene expression rhythm is present. Its damped profile is indicative of an unpregulation of the repressive light-input pathway (Beale et al. 2013). **f** Timed feeding entrains the peripheral core clocks of *P. andruzzii*, as *Per1* and *Clk1a* rhythms persist in the absence of the zeitgeber (Cavallari et al. 2011)

Of all the studies that examine rhythms in DD after entrainment (both as the natural condition of the caves and also the test condition of the defining feature of the circadian clock, i.e. the ability to free run), most show rhythmicity in the circadian range (Fig. 5). The clearest examples include six species of cave fishes: locomotor rhythms after LD entrainment in *Schistura jaruthani* (Fig. 5a), *Schistura spesei* (both Duboué and Borowsky 2012), *Rhamdia enfurnada* and *Trychomycterus* sp.2 (both Trajano et al. 2012); clock gene expression rhythms after LD entrainment in *Astyanax mexicanus* (Fig. 5e; Beale et al. 2013); and clock gene expression rhythms after feeding entrainment *Phreatichtys andruzzii* (Fig. 5f; Cavallari et al. 2011). Other examples demonstrate the fragile nature of studying locomotor rhythms and their analysis, by showing variation between individuals with rhythmic components seen in only a fraction of tested animals, including in *Nemacheilus evezardi* (Pati 2001), *Orconectes pellucidus* (Brown 1961; Jegla and Poulson 1968), and *Glyphiulus cavernicolous* (Koilraj et al. 2000).

The importance of experimental protocol in allowing an unambiguous interpretation of the chronobiology of a species is demonstrated by two papers in particular. In the first paper, Trajano et al. (2009) do not entrain the animals before attempting to measure locomotor activity in constant conditions—as a result, they see variation between individuals of *Rhamdia enfurnada*: some are rhythmic, others are not. However, a further paper in 2012 studied the same species in constant conditions after LD entrainment, and concluded that this cavefish possesses an entrainable circadian rhythm (Trajano et al. 2012). Similarly, a study of a cave millipede, *Bianulus lichtensteni*, conducted without entrainment, yielded ambiguous results (Mead and Gilhodes 1974). Only a fraction of individuals exhibited weak circadian rhythms, according to autocorrelation analysis, precluding reliable conclusions of the circadian phenotype of this species as a whole. Daily rhythms of activity are present in *Procambarus cavernicola* under entraining conditions, but the free-run condition the authors use is constant light, not constant darkness, limiting the usefulness of this study in comparisons (O-Martínez et al. 2004).

Only a few papers present evidence for a complete absence of rhythms in cave animals. These include the cave salamander, *Proteus anguinus* (Hervant and Mathieu 2000), two *Rhamdiopsis* genus cave catfishes (cited as *Taunayia* sp. in Trajano and Menna-Barreto (2000) and undescribed genus in Trajano et al. (2005), respectively), and the characiform *Stygichthys typhlops* (Trajano et al. 2012). However, caveats exist in all cases: *P. anguinus* was not entrained prior to free-run, and therefore it is difficult to conclude on the status of its rhythmic capabilities. Whilst the *Rhamdiopsis* species and *S. typhlops* were arrhythmic after LD entrainment, non-photic zeitgebers were not tested (though perhaps this was a deliberate choice after the fishes’ statistically significant response to LD cycles). Testing the role of non-photic zeitgebers is particularly pertinent given the results of a recent study of the Somalian cavefish, *Phreatichtys andruzzii* (Cavallari et al. 2011). Whilst this cavefish is arrhythmic after LD entrainment (Fig. 5b) with mutations in candidate circadian photoreceptors *tmt*-*opsin* and *melanopsin*, rhythms of locomotor activity and expression of core clock genes in the circadian range are exhibited after food entrainment (Fig. 5c, f; Cavallari et al. 2011). Nevertheless, cell culture based experiments give some suggestion that *P. andruzzii* exhibits rhythms in the infradian range (Cavallari et al. 2011). This is a possible condition for other cave animals: rhythms of a non-circadian range are observed in *Schistura oedipus* (Duboué and Borowsky 2012) and *Niphargus puteanus* (Blume et al. 1962). However, it would be interesting to see the responses of these animals to non-photic zeitgebers.

Both photic and temperature cycles were examined in one study of two species cave beetles (Lamprecht and Weber 1978). Unfortunately, the researchers did not study both zeitgebers on each species: *Aphaenops cereberus* was only exposed to LD cycles (of a very low magnitude, just 0.5 lx LD) and *Speonomus diecki* was only exposed to temperature cycles of 2.6 °C amplitude. Furthermore, in neither case was the beetle allowed to free run after entrainment, preventing proper circadian conclusions.

In summary, the majority of the evidence points to the retention of circadian rhythmic physiology in troglobites. It is worth noting that in these studies the major assay of circadian functionality is locomotor activity, which is sensitive to the type of analysis performed. Perhaps a clearer answer can be gained from an analysis of the molecular components. The molecular circadian clock is the core of rhythmic physiology, its transcription–translation feedback loop being the source of all output rhythms. Molecular studies on cave animals may reduce problems in assaying output rhythms and produce more consistent data. Demonstrating this is Beale et al.’s study on *A. mexicanus* cavefish (Beale et al. 2013). Whilst locomotor activity data is suggestive of circadian rhythms in this species (Fig. 5d; Erckens and Martin 1982; Beale et al. 2013), evidence from the molecular clock confirms that cavefish of this species retain an entrainable circadian clock (Fig. 5e; Beale et al. 2013).

**Fig. 6:**
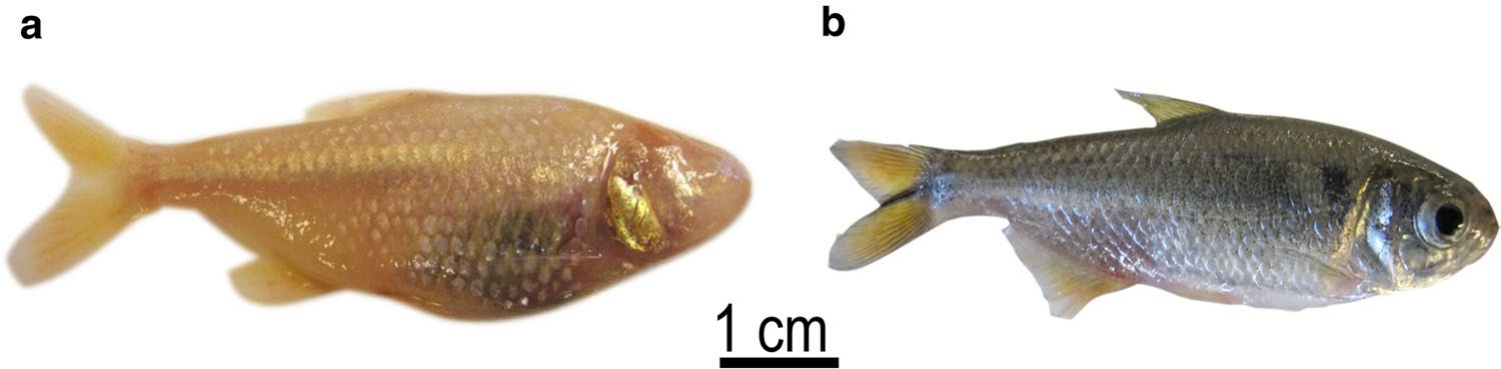
The Mexican tetra *Astyanax mexicanus* is a useful model for studying chronobiology as several distinct phenotypes are found within the same species complex. **a** Eyeless Pachón cave morph and **b** eyed surface morph

The recent circadian studies of *A. mexicanus* cavefish (Fig. 6) by Beale et al. (2013) and Moran et al. (2014) have also started to experimentally probe the adaptive significance of changes in the rhythmic physiology of this species complex as it adapted to subterranean habitats. Beale et al. (2013) measured the circadian coupled DNA repair pathways in surface and cave phenotypes, and suggested that the day-like semi-locked state of the cave variants’ circadian clocks likely helps sustain DNA repair under constant dark conditions (whereas this would have occurred during the day in the ancestral surface fish from which they evolved). Moran et al. (2014) measured the circadian metabolic rate under constant exercise conditions, and observed a significantly dampened diurnal increase in metabolic rate in a cave phenotype compared to the surface phenotype. Moran et al. (2014) suggested that the loss of the circadian rhythm in metabolism was an energy saving adaptation in an environment that did not require a rhythmic increase in energy metabolism to prepare for a day of foraging and predator evasion and that was likely food limited for at least part of the year. The next steps in this species will be to confirm the exact molecular alterations that have taken place in cave morphs during adaptation to the cave environment, and the degree to which physiological pathways have been altered or decoupled from the molecular genetic clocks.

Recent work in sequencing the genomes of cavefish with closely related surface populations is giving insights into the genetic changes during cave adaptation (McGaugh et al. 2014; Yang et al. 2016). In the cavefish *Sinocyclochelus anshuiensis* Yang et al. (2016) find a truncation in *skp1*, an important component of the E3 ligase SCF1 which regulates the clock through the targeting of proteins for degradation (Busino et al. 2007), suggesting that circadian function is disrupted. This truncation is not found in the surface-dwelling form. They also report the downregulation of circadian genes in the cavefish, though the cave form does express the non-visual opsins and candidate circadian photoreceptors *melanopsin* (*opn4xa* and *opn4xb*) and *tmt*-*opsin2a* (Yang et al. 2016). Unfortunately they do not specify which circadian genes are downregulated and they do not say at which time point the fish were sampled, so the functional status of the molecular circadian clock in these cavefish is somewhat unknown.

In the light of these studies, it would be interesting to re-examine the cave animals of historical studies using modern molecular and physiological techniques to explore the variation and consistency in rhythmic physiology between independently derived cave species.

## What can be gained in our understanding of the evolution of physiological rhythmicity by comparing animals from rhythmic and arrhythmic habitats?

Given the widespread distribution of circadian systems between organisms (and within the cells of those organisms), together with the similarity of their mechanisms and their influence on a great deal of an organism’s physiology, it is not surprising that extensive efforts have been directed at understanding their adaptive significance. It has been suggested that rhythmic physiology is an adaptation for a rotating planet, implying the importance of the external environment in the fitness advantage given by the clock. However, rhythmic physiology is not solely concerned with synchronising with the external world, since a variety of internal physiological processes, including metabolism, need to be organised temporally to avoid conflict. Two hypotheses for the evolutionary advantage of circadian clocks are derived from these arguments: extrinsic and intrinsic organisation, or the ‘Day Outside’ and the ‘Day Within’ (Pittendrigh 1993; Sharma 2003; Yerushalmi and Green 2009; Vaze and Sharma 2013). These hypotheses are not mutually exclusive: it is possible that selection for intrinsic organisation originated through order forced upon cell processes by the regular variation in temperature and UV and visible light, and therefore is a result of the cyclical external world. Animals from arrhythmic habitats offer the chance to directly test the first hypothesis and learn about the adaptive value of rhythmic physiology at the Earth’s surface, and why rhythmicity evolved. By removing regular rhythmicity from the external environment, one removes any extrinsic evolutionary pressure on the circadian clock system. If rhythmic physiology persists or has originated in these habitats, it may have more to do with providing internal temporal order—an intrinsic evolutionary pressure, and giving evidence supporting the Day Within hypothesis.

Therefore, a great deal of information on the adaptive significance of clocks in animals can come from studies that focus on the association between the environment and circadian phenotype, especially in habitats with little to no daily variation or in habitats with extreme annual variation in photoperiod, such as the polar regions. It is likely that populations that have evolved under these environmental conditions should exhibit variation in their circadian phenotypes reflecting those conditions, providing a very strong test for the two hypotheses of the fitness advantage offered by the circadian mechanism.

Multiple approaches have been taken to examine the fitness advantage of rhythmic physiology, but have predominantly focused on the value of the circadian clock with respect to a rhythmic environment, including experimental manipulation of circadian phenotype and/or environment and observations of correlation between variation in environment and variation in circadian properties. Recent reviews have outlined the strong evidence from these studies that clocks give a selective advantage to organisms (see Vaze and Sharma 2013; Yerushalmi and Green 2009), so following here is just a summary.

### Evidence for the evolutionary advantage of rhythmic physiology

Compelling experimental evidence that circadian clocks improve the fitness of organisms is relatively sparse. Some of the clearest evidence comes from selection experiments performed in plants, longevity studies in animals with disrupted circadian rhythms, and in studies examining the property of circadian resonance, the degree of matching between the endogenous period of the clock and the period of the external environment. When arrhythmic and wild-type *Arabidopsis thaliana* are grown under extreme short-day conditions, arrhythmic plants are less viable than the wild-type plants, with less than 5 % of seedlings surviving for 4 weeks (Green et al. 2002). Green and colleagues suggested that the lack of a clock puts the plants at a disadvantage as arrhythmic plants fail to anticipate the day, as seen in the delay in expression in clock gene transcripts compared to wild-type in LD cycles.

Survival comparisons of rhythmic and arrhythmic animals also show effects of circadian rhythm disruption on fitness. DeCoursey et al. (2000) investigated the survival of chipmunks in semi-natural conditions where animals had been subjected to SCN lesions. After an initial period where both sham- and SCN-operated groups showed higher mortality than non-operated, the survival curves stabilised and a gradual trend of higher mortality in SCN-lesioned animals was observed. After 80 days, SCN-lesioned animals showed losses of 80 %, compared to 50 % in sham controls. The authors speculate that the increased night-time restlessness in lesioned animals may have attracted predators to the burrows, resulting in the loss of more of the population. Though survival is not a direct measure of fitness, this result is suggestive of a fitness value to the circadian clock, since most of the SCN-lesioned animals would not survive long enough to produce young.

Further evidence comes from circadian resonance studies, where organisms have had the relationship altered between their endogenous clock period and the environmental period. The circadian resonance hypothesis states that fitness is enhanced when the endogenous period and external cycles are closely matched, or ‘resonant’, and within the range in which the endogenous rhythms can be entrained to the external cycles. Organisms with discordant clocks suffer a fitness loss. In plants, short- and long-period *A. thaliana* mutants, sown under 20 and 28 h T-cycles, were measured for rosette diameter, chlorophyll content, leaf number and total mass. The short-period mutant performed better under 20 h T-cycles whereas the long-period mutant performed better under 28 h T-cycles; matching endogenous period with environmental period significantly improves the plant’s output (Dodd et al. 2005). Similar results have been reported for cyanobacteria, where resonant strains outcompete non-resonant strains (Ouyang et al. 1998; Woelfle et al. 2004).

Pittendrigh first reported on the effects of matching external and endogenous period on animal lifespan, where flies raised in photoperiods divergent from 24 h lived shorter than those on 24-h photoperiod (Pittendrigh and Minis 1972). Comparable results were reported for blowflies by Aschoff (Saint Paul and Aschoff 1978). In a circadian resonance study analogous to that performed in *A. thaliana*, wild-type, short- and long-period *Drosophila* mutants show mild lifespan reductions when the endogenous period does not match environmental period, though wild-type flies have longer lifespans than mutants in all tested photoperiods (Klarsfeld and Rouyer 1998). In mammals, studies on the period length mutants of hamsters revealed that, when kept on 24-h cycles, mutants show small reductions in life span and increased incidences of disease, with heterozygous (*tau*/+) mutants showing the greatest effects (Hurd and Ralph 1998; Martino et al. 2008).

Whilst indicative, longevity, growth, and developmental rates do not directly assess the fitness advantage of the circadian clock in animals. Using *tau* period mutant mice, Spoelstra and colleagues directly assessed the fitness advantage of a circadian clock in semi-natural conditions (Spoelstra et al. 2016). When kept in constant darkness, heterozygous and homozygous *tau* mutant mice free-run with circadian period lengths of about 22 and 20 h, respectively, resulting in a circadian dissonance to the natural 24-h day. Spoelstra and colleagues recorded long-term behaviour, survival rates and allele frequency of mixed populations of wild-type, heterozygous and homozygous mutants released in Mendelian ratios to external pens (Spoelstra et al. 2016). Homozygous mice exhibit significantly increased mortality and the lowest probability of survival of the three groups. In addition, new generations of mice born during the course of the experiment contained fewer than expected numbers of homozygotes. In total, the allele frequency of the *tau* allele decreased from 50 % at the start of the experiment to 20 % 14 months later, indicating a strong selection against the *tau* allele, and hence against the non-resonant period circadian clock.

These experiments demonstrate the value of the clock with respect to the external environment and suggest that the clock provides a fitness advantage to an organism in rhythmic environments. What can be said for the circadian clock in arrhythmic environments? For cyanobacteria kept in constant light, strains with a functional clock are outcompeted by strains with a mutant clock, suggesting that the clock is of no benefit or even detrimental in constant conditions (Woelfle et al. 2004). By contrast, *Drosophila* raised for 700 generations in constant light retain entrainable circadian rhythms, which the authors suggest is indicative of the circadian clocks having an intrinsic adaptive value (Paranjpe et al. 2003). Of course, animals from arrhythmic habitats are natural examples of these experimental conditions, and provide another method to test the Day Within hypothesis.

In polar regions, though evidence for a functional molecular clock is not yet conclusive, the plastic state of rhythmic behaviour in polar animals across the seasons (Steiger et al. 2013) suggests that synchrony with the external environment is not the primary role for the circadian clock in these animals, though any cost associated with a lack of synchrony with external environment should be assessed by systematically comparing such individuals with those which are in synchrony with the environment. However, this does not imply that circadian clock is not adaptive in arrhythmic conditions. Polar animals could lose or weaken the expression of some external outputs of the circadian clock, but retain the clock for its role within the organism. Support for this position can be found in an animal from a subterranean habitat, the blind mole rat, *Spalax ehrenbergi*. Weak coupling between this organism’s locomotor activity and its internal clock similarly suggests that coordination of activity with the external world might not be important to this organism, though it still perceives daily and seasonal temporal cycles even underground (Nevo 1998). Further evidence is found in the deep sea and caves. Pineal melatonin rhythms are present in deep-sea fishes (Wagner et al. 2007), and entrainable locomotor activity and molecular circadian clock rhythms are present in multiple species of cave animals (Cavallari et al. 2011; Duboué and Borowsky 2012; Trajano et al. 2012; Beale et al. 2013), suggesting that the clock has not been lost even in arrhythmic environments.

That rhythmicity persists in arrhythmic habitats leads us to question the value of this generated rhythmicity —something other than simply coordinating an animal’s physiology with the external environment. It is suggestive that there is pressure from the “Day Within” to maintain temporal organisation in the organism. However, it is worth noting an alternative possibility: that circadian rhythms in arrhythmic environments are vestigial with species retaining differing degrees of functionality.

This alternative explanation is less favoured for at least three reasons. Firstly, enough time has passed for significant trait loss during isolation underground—characteristics such as pigmentation and vision are lost in almost all obligate subterranean animals—but circadian rhythms do not seem to be one of them. Secondly, circadian rhythms are in some form retained across many species, despite vast differences in evolutionary histories and environmental pressures, and invasion time seems to be unrelated to circadian function. Though estimates of invasion of the subterranean habitats are broad, circadian rhythms are retained in cave species that invaded upwards of 1 million years ago (mya): 1.12–4.6 mya for *A. mexicanus* cave populations (Strecker et al. 2004; Ornelas-García et al. 2008; Bradic et al. 2012; Gross 2012; Espinasa and Espinasa 2016); 1.4–2.6 mya for *P. andruzzii* cavefish (Colli et al. 2009). By contrast, rhythms are apparently absent in *Proteus anguinus* despite its relatively recent (10,000 years ago) invasion of the caves (Hervant and Mathieu 2000; Sket 1997; Goricki and Trontelj 2006). Thirdly, in a genetic mechanism such as the circadian clock, it is likely that one or more of the genes involved would exhibit a degree of non-functionality if the clock were vestigial and, with the exception of the truncated E3 ligase component *skp1* in *S. anshuiensis* (Yang et al. 2016), this is not the case in *A. mexicanus*, *P. andruzzi*, *S. ehrenbergi* or *Ptomaphagus hirtus* (Avivi et al. 2002, 2004; Cavallari et al. 2011; Friedrich et al. 2011; Beale et al. 2013). Moreover, even if some components were mutated, redundancy in the molecular network (especially in teleosts with multiple copies of genes) may be a buffer to complete loss of function.

We are drawn back to the Day Within. Clocks are involved in a vast amount of cellular physiology including metabolism and energy storage (Green et al. 2008; Asher and Schibler 2011; Eckel-Mahan and Sassone-Corsi 2013); regulation of the DNA repair pathways (Sancar et al. 2015); cell division (Johnson 2010; Masri et al. 2013; Tamai and Whitmore 2015); and, in plants, separation of photosynthesis and nitrogen fixation reactions (Berman-Frank et al. 2001). These independent roles do not require the complex free-running system of the circadian clock, but as these loops cooperate and reinforce one another, they increase the resistance to external environmental fluctuation and give clocks a selective advantage due to intrinsic organisation (Brown et al. 2012). The data from studies of animals from arrhythmic environments support this hypothesis—data that can only come when the rhythmic environment is removed from the equation.

There is one final caveat: have we found anywhere on Earth that is truly outside the influence of the sun and therefore, have we found a true arrhythmic condition to test the fitness advantage of rhythmic physiology? Even the deep sea, at thousands of metres depth, feels the influence of the sun (and moon) through the movement of animals in the water column and through the movement of water due to tides. Deep caves, isolated from the external world, may still have links to the outside world, whether through the slow movement of air through cave complexes or the shuttling of organisms such as bats from the entrance to the deep. Nevertheless, these studies of arrhythmic habitats have great power to inform our understanding of the importance of rhythmic physiology. Perhaps our best hope is in organisms that are independent of the energy given by the sun, such as those that exist on the deep-sea hydrothermal vents. Is there a swathe of organisms here that have never evolved what appears to be a universal feature of life on Earth?

Rhythmic physiology is a fundamental structuring element of biology and a deeper comprehension of this trait will impact us in many ways, from fields as varied as human and domesticated animal health, to subsurface ecosystem function, human space exploration and understanding life on other worlds. Given advances in technology, especially biotechnology, we are increasingly empowered to study biological rhythms in animals from dark places, and if we are to properly understand the evolution and significance of rhythmic physiology we need to extend our view beyond the small, lit envelope we humans inhabit.

## Abbreviations

LD: Light–dark
DD: Constant darkness
ZT: Zeitgeber time
CT: Circadian time
TTFL: Transcription–translation feedback loop
